# Human decellularized bone scaffolds from aged donors show improved osteoinductive capacity compared to young donor bone

**DOI:** 10.1371/journal.pone.0177416

**Published:** 2017-05-15

**Authors:** Christopher A. Smith, Tim N. Board, Paul Rooney, Mark J. Eagle, Stephen M. Richardson, Judith A. Hoyland

**Affiliations:** 1 Divsion of Cell Matrix Biology and Regenerative Medicine, School of Biological Sciences, Faculty of Biology, Medicine and Health, University of Manchester, Manchester, United Kingdom; 2 Wrightington Hospital, Wigan, United Kingdom; 3 National Health Service (NHS) Blood and Tissue Transplant Services, Speke, Liverpool, United Kingdom; 4 NIHR Manchester Musculoskeletal Biomedical Research Unit, Central Manchester Foundation Trust, Manchester Academic Health Science Centre, Manchester, United Kingdom; University of Sheffield, UNITED KINGDOM

## Abstract

To improve the safe use of allograft bone, decellularization techniques may be utilized to produce acellular scaffolds. Such scaffolds should retain their innate biological and biomechanical capacity and support mesenchymal stem cell (MSC) osteogenic differentiation. However, as allograft bone is derived from a wide age-range, this study aimed to determine whether donor age impacts on the ability an osteoinductive, acellular scaffold produced from human bone to promote the osteogenic differentiation of bone marrow MSCs (BM-MSC). BM-MSCs from young and old donors were seeded on acellular bone cubes from young and old donors undergoing osteoarthritis related hip surgery. All combinations resulted in increased osteogenic gene expression, and alkaline phosphatase (ALP) enzyme activity, however BM-MSCs cultured on old donor bone displayed the largest increases. BM-MSCs cultured in old donor bone conditioned media also displayed higher osteogenic gene expression and ALP activity than those exposed to young donor bone conditioned media. ELISA and Luminex analysis of conditioned media demonstrated similar levels of bioactive factors between age groups; however, IGF binding protein 1 (IGFBP1) concentration was significantly higher in young donor samples. Additionally, structural analysis of old donor bone indicated an increased porosity compared to young donor bone. These results demonstrate the ability of a decellularized scaffold produced from young and old donors to support osteogenic differentiation of cells from young and old donors. Significantly, the older donor bone produced greater osteogenic differentiation which may be related to reduced IGFBP1 bioavailability and increased porosity, potentially explaining the excellent clinical results seen with the use of allograft from aged donors.

## Introduction

The number of surgical procedures requiring bone graft to promote bone repair/ regeneration is set to substantially increase in the near future [1]. Importantly, this increase will include the number of procedures on younger patients. Thus, there is a need to improve osseointegration of prosthetics, increasing their longevity, thereby reducing the need for revision surgery and further impact on the patient’s life. While autograft is considered the “gold standard” for bone regeneration, the issues of availability and donor site morbidity have meant allografts are more commonly used [2]. However, allograft displays inferior osteogenic qualities whilst also raising concerns regarding immune response and pathogen transfer [3, 4]. Bone tissue engineering, using autologous mesenchymal stem cells (MSCs) combined with osteogenic supportive scaffolds, is often proposed as an alternative to the use of allograft and has the potential to address these issues. However, although there are multiple synthetic scaffolds available, they display inadequate de novo bone formation and lack the innate microenvironment and material qualities associated with native bone, thus limiting their potential for clinical application [5]. To utilise the innate qualities of bone in a safe manner, decellularization technologies are employed to remove all cells and potentially harmful immunogenic components from the allograft bone. These processes aim to produce scaffolds which still benefit from the complex microenvironmental properties of native tissue [6, 7]. However, such methodologies often take time, are laborious, produce small fragments at inadequate volumes for use in large defect sites, and commonly use chemicals, which may impact on the osteoinductive capacity and mechanical stability.

Previously, we have detailed the use of a novel wash process to rapidly produce large volumes of biocompatible, structurally stable, and osteoinductive human bone biological scaffolds [8], which meet the criteria of a decellularized material (as defined by Crapo et al [9]). Importantly, this decellularized scaffold is also osteoinductive to seeded bone marrow MSCs (BM-MSCs), and therefore suitable as a biological scaffold in bone tissue engineering. However, as this decellularized material is donor-derived and a large majority of bone allograft is currently retrieved from aged (> 70 years) individuals, it is important to ascertain whether the osteoinductive capacity of such material is affected by donor age.

Whilst they are many factors that may affect bone healing such as weight, disease and gender, aging has been shown to have an effect on bone architecture (topography, pore size and porosity)[10, 11], mineral crystallinity and extracellular matrix (ECM) composition, all of which impact on its osteoinductive capacity [12, 13]. As such, the impact of donor age, specifically on its potential osteoinductive make it a dominant factor in determining feasibility of use in clinic.

Existing studies demonstrate conflicting findings as to whether the age of the bone donor does [14, 15] or does not [16–19] influence the bone’s osteoinductive potential. Such research has, however, been restricted to powdered demineralised bone matrix (DBM), or unprocessed fresh-frozen allograft, with the majority of studies being undertaken in rodent models. As such these studies fail to address how age-related changes in structure or composition of human bone ECM in decellularized biological scaffolds may influence its potential as a scaffold for MSC-based bone regeneration.

As the age of both the recipient and the donor of the decellularized allograft bone used may have an impact on the hosts’ response, particularly osseointegration, and clinical outcome, it is essential to evaluate how donor age may impact on the osteogenic activity of the allograft. Specifically, in using autologous BM-MSCs, the age of the donor bone is the only clinically controllable factor. As such, this study aims to assess the effect of bone donor age on the osteogenic differentiation and activity of young and old BM-MSCs seeded on the decellularized bone allograft, analysing the known osteoinductive qualities of structure, mineral content and soluble factors that may differ between the two age groups.

## Materials and methods

### Comparison of young and old donor MSCs seeded on young and old decellularized bone

Human femoral heads were obtained by National Health Service Blood and Transplant, Tissue Services (NHSBT), under ethical approval, with written informed consent from live young (≤50 years, 34–49, N = 7) and old (≥70 years, 72–84, N = 9) non-osteoporotic donors undergoing primary hip replacement surgery due to osteoarthritis, representative of routinely used allogenic bone graft material. Femoral heads were decellularized and processed to 1cm^3^ bone cubes utilising our previously described novel wash process [8].

Bone marrow was obtained with relevant National Research Ethics Service and University of Manchester approvals and written informed consent from young (≤50 years, 44–49, N = 4) and old (≥70 years, 72–81, N = 3) patients undergoing non-osteoporotic, osteoarthritis related hip replacement. BM-MSCs were then isolated in line with established methodology and were cultured as previously described in standard medium [8]. At passage 3, 0.5x10^6^ cells from individual cell samples suspended in 125μl of media were seeded separately onto decellularized bone cubes from 3 young and 3 old bone donors, to produce cell donor matched, seeded young and old donor bone samples with cell and bone samples tracked individually. Samples were incubated for 1 hour (37°C, 5% CO_2_) and then non-adherent cells removed by a PBS wash and centrifugation (500xg, 5 minutes). The seeded bone cubes were subsequently cultured at 37°C in 2ml of osteogenic differentiation medium [8] for 28 days, with media changed every 3 days. At time-points 0, 14 and 28 days metabolic activity was assessed using 5% alamarBlue in osteogenic medium to minimise effect to culture conditions, before time-point relevant samples were centrifuged dry (500xg, 5 minutes) and used to assess gene expression and alkaline phosphatase (ALP) activity. AlamarBlue metabolic activity readings were normalised to non-seeded bone in osteogenic medium to account for any effect this may have had on resazurin reduction.

### Gene transcription analysis

To isolate RNA, bone cubes were transferred to 1mL TRIzol reagent (Ambion), which was then removed from the sample by centrifugation (500xg, 5 minutes). RNA was extracted as previously described [8], quantified by Nanodrop and reverse transcribed using a High Capacity cDNA Reverse Transcription kit (Life Technologies). Quantitative real-time polymerase chain reaction (QRT-PCR) was performed, using the Lumino-Ct qPCR ReadyMix (Sigma Aldrich) prepared with primers and FAM-BHQ1 probes for the osteogenic genes: runt related transcription factor 2 (RUNX2), osteopontin (OP) and osteocalcin (BGLAP) (OC) (all primers and probes from Sigma Aldrich; Table 1). Data was normalised to the reference gene mitochondrial ribosome protein 19 (MRPL19) and results displayed relative to day 0 controls (2^-ΔΔCT^).

**Table 1:** Primer and probe sequences for use in QRT-PCR.

Target Gene	Accession ID (Gene bank)	Forward primer seq.	Reverse primer seq.	Probe sequence	Working conc. of primer (nM)
MRPL19	NM_014763	CCA CAT TCC AGA GTT CTA	CCG AGG ATT ATA AAG TTC AAA	CAA ATC TCG ACA CCT TGT CCT TCG	900
RUNX2	NM_001024630	CGC TGC AAC AAG ACC	CGC CAT GAC AGT AAC C	TGG CCT TCA AGG TGG TAG CCC TC	900
Osteopontin (OPN)	NM_000582	CTG ACA TCC AGT ACC CTG	CAG CTG ACT CGT TTC ATA	CTG TCC TTC CCA CGG CTG TC	600
Osteocalcin (OC)	NM_199173	CCG CAC TTT GCA TCG	GCC ATT GAT ACA GGT AGC	CCA GGC AGG TGC GAA GCC C	600

### ALP enzyme activity

BM-MSCs from young (N = 3) or old (N = 3) cell donors were individually seeded onto a decellularized bone cube from both 1 of 3 young and 1 of 3 old bone donors to produce a total of 12 samples, and cultured for 28 days. Samples were lysed in 0.5% Triton X-100 containing 5mM MgCl_2_, then centrifuged (10,000xg, 10 minutes, 4°C) to remove bone particles. Triplicate, 50 μl aliquots of each sample were mixed 1:1 with ALP substrate solution (5 mM p-NPP [Sigma], 150mM AMP buffer [Sigma] and 5 mM MgCl_2_), and measured for absorbance at 420nm immediately, and again after a 30-minute incubation at 21°C. Additional triplicate, 50μl aliquots were analysed with Picogreen to determine DNA content for normalisation. Enzyme activity was calculated as displayed where t30 = absorbance at 30 minutes, t0 = absorbance at 0 minutes, t = time period and v = volume measured

$$\text{Enzyme activity}=\frac{\left(\text{t}30-\text{t}0\right)÷\left(\text{v x t}\right)}{\text{DNA }\left(\text{ng}\right)}$$

Enzyme activity was normalised to day 0 controls.

### Analysis of the structural and material components for young and old donor bone

#### μCT, X-ray diffraction and X-ray fluorescence

For microcomputed tomography (μCT) assessment of the decellularized bone cubes, samples from 4 young (≤50 years, range 34–49 years, n = 16 cubes) and 3 old donors (≥70 years, range 72–75 years, n = 13 cubes) taken from below the femoral head growth plate were rotated 180°, and images taken at 0.9° intervals (70kV; 140μA, 4 seconds), with a spatial resolution of 14.6μm per pixel (SkyScan 1172 and associated software NRecon, CTAn). Images were reconstructed and global thresholding used to produce binary images. For each sample, a volume of interest (VOI) (designated tissue volume [TV]) was selected and standard 3D analysis.

Decellularized bone cubes from young (N = 3) and old donors (N = 3) were assessed using X-ray diffraction (XRD), measured in 2θ with step size: 0.008 2θ/second, and scan range: 10° to 70° (Phillips X’Pert-MPD). Samples were subsequently analysed by X-ray fluorescence (XRF) (Minipal 4 EDXRF) to measure calcium to phosphorus ratio (Ca/P).

### Assessment of osteoinductive capacity of decellularized bone conditioned media

#### Production and use of decellularized bone conditioned media

Samples of decellularized bone from young (N = 3) and old (N = 3) bone donors were snap-frozen, powdered and incubated in standard medium at 0.05g/ml, alongside standard medium for use as a control for 72 hours at 37°C as previously described [8], The resulting solution was filtered to 0.22μM to remove small bone fragments and produce decellularized bone conditioned media (DBCM).

#### Effect of DBCM on MSC proliferation and differentiation

Young donor BM-MSCs were seeded at 3x10^4^/cm^2^ in 96 well plates. After 24 hours cells were cultured in all 6 DBCMs, from young (N = 3) and old (N = 3) bone donors, as well as standard medium. At 0 and 14 day time-points cell metabolic activity and osteogenic gene expression was assessed. At the 14 day endpoint, additional samples for lactate dehydrogenase (LDH), Picogreen and ALP enzyme activity were lysed in 200μl dH_2_O with three freeze-thaw cycles. To determine LDH concentration, a 100μl aliquot of each lysed sample was mixed with 100μl of LDH reagent (Sigma), and then incubated at room temperature for 30 minutes. Samples were measured for absorbance at 485nm/620nm.

#### ALP staining

In combination with ALP activity, in situ ALP staining was undertaken to quantify ALP presence, as ALP may have been less active not less abundant. Cell monolayers (N = 3) were fixed in 4% formal buffered saline for 1 minute, washed in PBS, and then incubated for 10 minutes in 250μl of BCIP/NBT solution (Sigma). Images were captured using light scanner, and analysed with ImageJ. Staining intensity was measured by densitometry. Images were converted to grey scale, then average pixel greyscale pixel values measured, and expressed as percentage black, whereby clear is 0% and black is 100%.

### Assessment of DBCM growth factor composition

A magnetic bead Luminex assay (Bio-techne) was constructed for the detection of BMPs 2, 4 & 9, IGFBP-1, IGFBP-3 and osteopontin in the DBCM. The data was analysed using Bio-Plex (BioRad). Sandwich ELISA assays were used to detect human IGF-I (Bio-techne) and IGF-II (Abnova) in the DBCM. All samples were run in triplicate and assays undertaken using suppliers methodology.

### Statistical analysis

All statistical analysis was conducted in GraphPad Prism software, using a non-parametric Mann-Whitney U test. Statistical significance was accepted for p≤0.05. Gene transcription analysis, ALP activity, ALP staining and growth factor analysis were analysed for statistical comparison of samples at each timepoint with day 0 controls, and between conditions at the same time point. Statistical analysis of μCT data was performed to compare grouped young and old samples for each parameter tested.

## Results

### Bone donor age influences osteogenic differentiation of seeded young and old donor BM-MSCs

**Fig 1:**
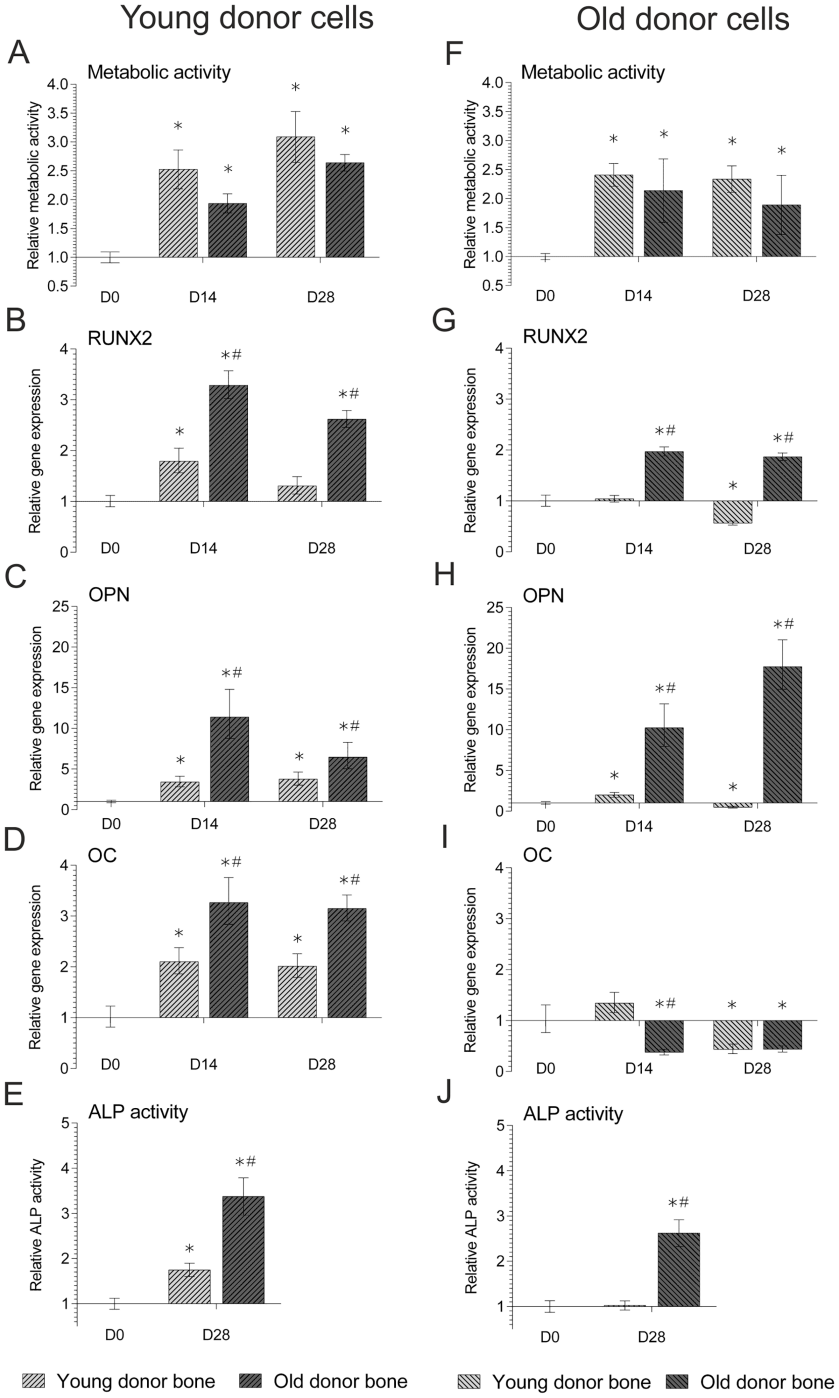
AlamarBlue metabolic activity, QRT-PCR gene expression, and ALP activity in BM-MSCs isolated from young (≤50 years)(a-e) and old (≥70 years)(f-j) donors seeded on decellularized bone scaffolds from either young (≤50 years) or old (≥70 years) bone donors. Relative metabolic activity (a, f) was normalised to non-seeded bone controls and day 0 readings whilst relative gene expression of osteogenic markers RUNX2 (b, g), OPN (c, h) and OC (d, i) were normalised to the reference gene MRPL19 and day 0 controls (2^-ΔΔCT^). Relative ALP enzymatic activity (e, j) was normalised to non-seeded bone controls, DNA content and day 0 readings. Data represent mean ± SE. * indicates significant difference with respect to 0d control (p≤0.05). # indicates significant difference between young and old samples at the same time-point (p≤0.05).

Picogreen analysis of seeded cells indicated no significant difference in the DNA content of cells seeded on the young or old donor allograft (436.6 ng ±81 SD and 357.9 ng ±173.1 SD respectively, p = 0.3). Results indicated that BM-MSCs isolated from either young or old cell donors remained metabolically active when seeded on decellularized bone cubes with significant increases in metabolic activity at day 14 and 28, (Fig 1A and 1F). Young donor BM-MSCs underwent osteogenic differentiation when cultured on decellularized bone from either young or old bone donors, displaying significant increases in RUNX2 (Fig 1B), OPN (1C) and OC (1D) gene expression in comparison to day 0, as well as significant increases in ALP activity (Fig 1E). However, at both time-points, young donor BM-MSCs cultured on old donor bone showed significantly higher gene expression of all target osteogenic genes and ALP activity, than those cultured on young donor bone.

Old donor BM-MSCs cultured on old donor bone displayed significant increases in RUNX2 (Fig 1G) and OPN (Fig 1H), and whilst no old donor cells displayed a significant increase in OC (Fig 1I), there was a significant increase in ALP activity (Fig 1J), in comparison to values at day 0. Additionally, old donor cells cultured on old donor bone displayed significantly higher gene expression and ALP activity than those cells cultured on young donor bone, which displayed no significant increase in any osteogenic gene other than OPN, or ALP activity.

### Does bone architecture differ between young and old donor decellularized bone?

**Fig 2:**
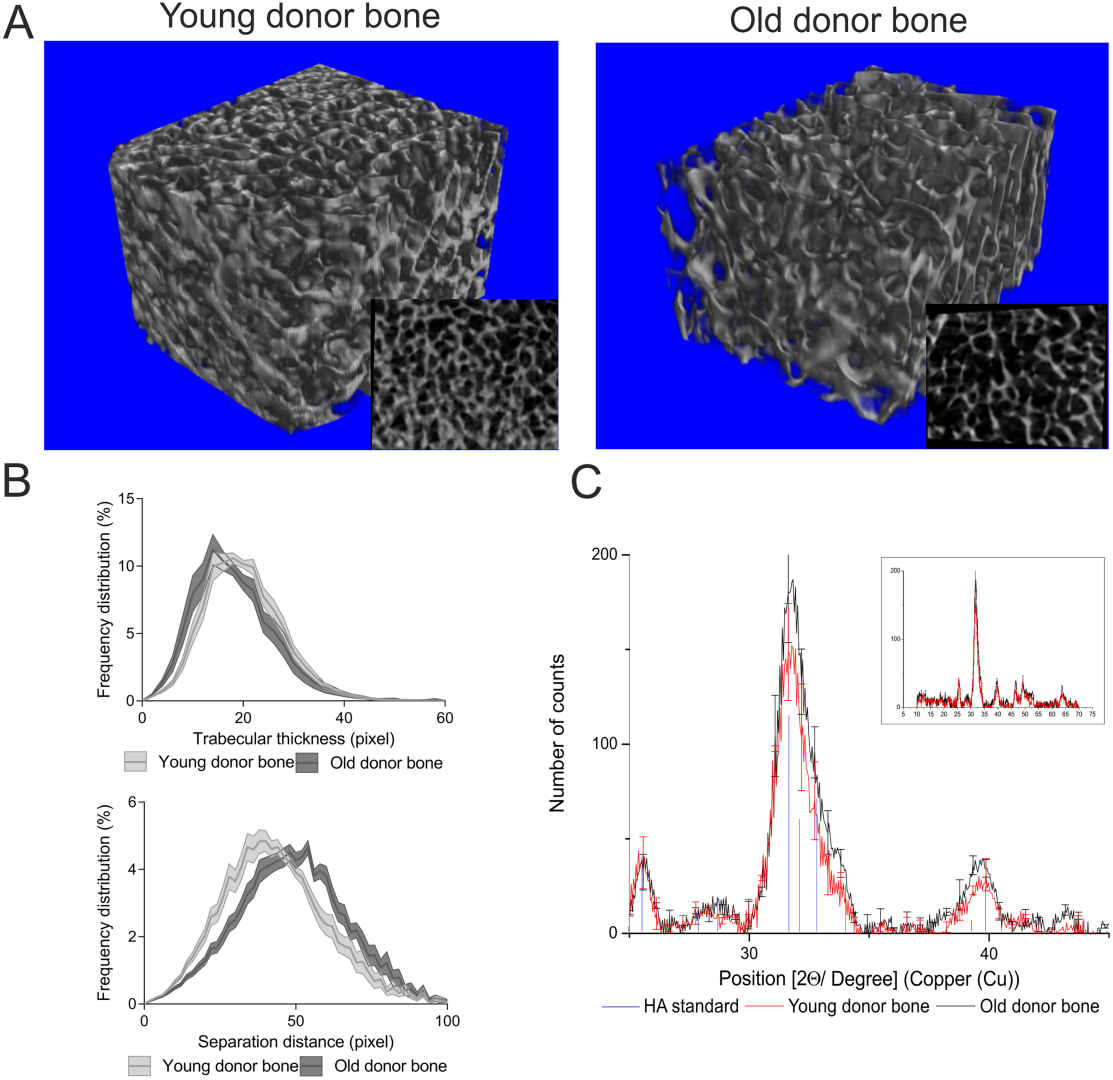
Analysis of bone cube architecture by μCT 3D modelling, and mineral crystallinity by XRD signal distribution analysis. Bone cube 3D models including insert of μCT cut-through (a), and frequency distribution plots for trabecular separation and thickness (b), for young (≤50 years) and old (≥70 years) were produced from donor bone analysed by μCT for a 180° rotation with images taken every 0.9°. Data displayed in pixels (1 pixel = 14.62 μM). XRD patterns of signal diffraction in young (≤50 years) and old (≥70 years) donor decellularized bone scaffold including exploded view insert (N = 3 for both), are displayed as line charts with error bars every 15 positions, for young (red) and old (black) donor bone, and the hydroxyapatite standard (HA JCPDS 74–0565), displayed as a line drop (blue). All measurements are in deflection angle (2-Theta [2Ɵ]).

μCT 3D analysis demonstrated differences between the architecture of young and old donor decellularized bone structures (Table 2 and Fig 2) (see S1 Table in supporting information for individual sample data). Young donor decellularized bone was significantly denser (BV/TV) and had a higher bone surface density (BS/TV) than old donor decellularized bone. The increase in porosity of old donor bone compared to young donor bone was evident in 3D models (Fig 2A), and was supported by a decrease in trabecular number (Tb.N) and an increase in the mean and distribution of trabecular separation (Tb.Sp), although there was no change in trabecular thickness (Tb.Th) (Fig 2B).

**Table 2:** Values, difference between groups and significance values for μCT microarchitecture analysis of young and old donor bone.

Parameter	Index	Young	Old	Difference	P value
Percent bone volume	BV/TV	32.80	25.44	7.36	**0.01**
Bone surface / volume ratio	BS/BV	0.18	0.21	0.03	0.14
Bone surface density	BS/TV	0.06	0.05	0.01	**0.01**
Trabecular pattern factor	Tb.Pf	0.01	0.03	0.02	0.06
Trabecular thickness	Tb.Th	20.21	18.37	1.83	0.22
Trabecular number	Tb.N	0.016	0.013	0.0025	**0.02**
Trabecular separation	Tb.Sp	43.54	48.96	5.42	**0.04**
Open porosity	Po(op)	67.20	74.55	7.36	**0.04**
Connectivity density	Conn.Dn	1.56E-05	1.15E-05	4.09E-06	**0.03**

Besides an increased porosity, there was a significant change in the architecture between young and old bone (Fig 2A). Old donor bone displayed a poorly interconnected trabecular lattice in comparison to young donor whilst displaying a decreased Connectivity (Conn.Dn).

### Does the mineral composition of the decellularized bone differ with age?

Results of mineral phase crystallinity analysis by XRD indicated that bone samples from either age group contained hydroxyapatite in their structure, with peaks comparable to the hydroxyapatite standard, with no significant difference between them (Fig 2C). Further comparison of the mineral composition of young and old bone samples by μCT and XRF, displayed no significant differences in the bone mineral density (2.1±0.14 and 1.9±0.15; respectively) or Ca/P ratio (1:5±2 and 1:3.6±1; respectively).

### Is the osteoinductive capacity of decellularized bone conditioned media influenced by bone donor age?

**Fig 3:**
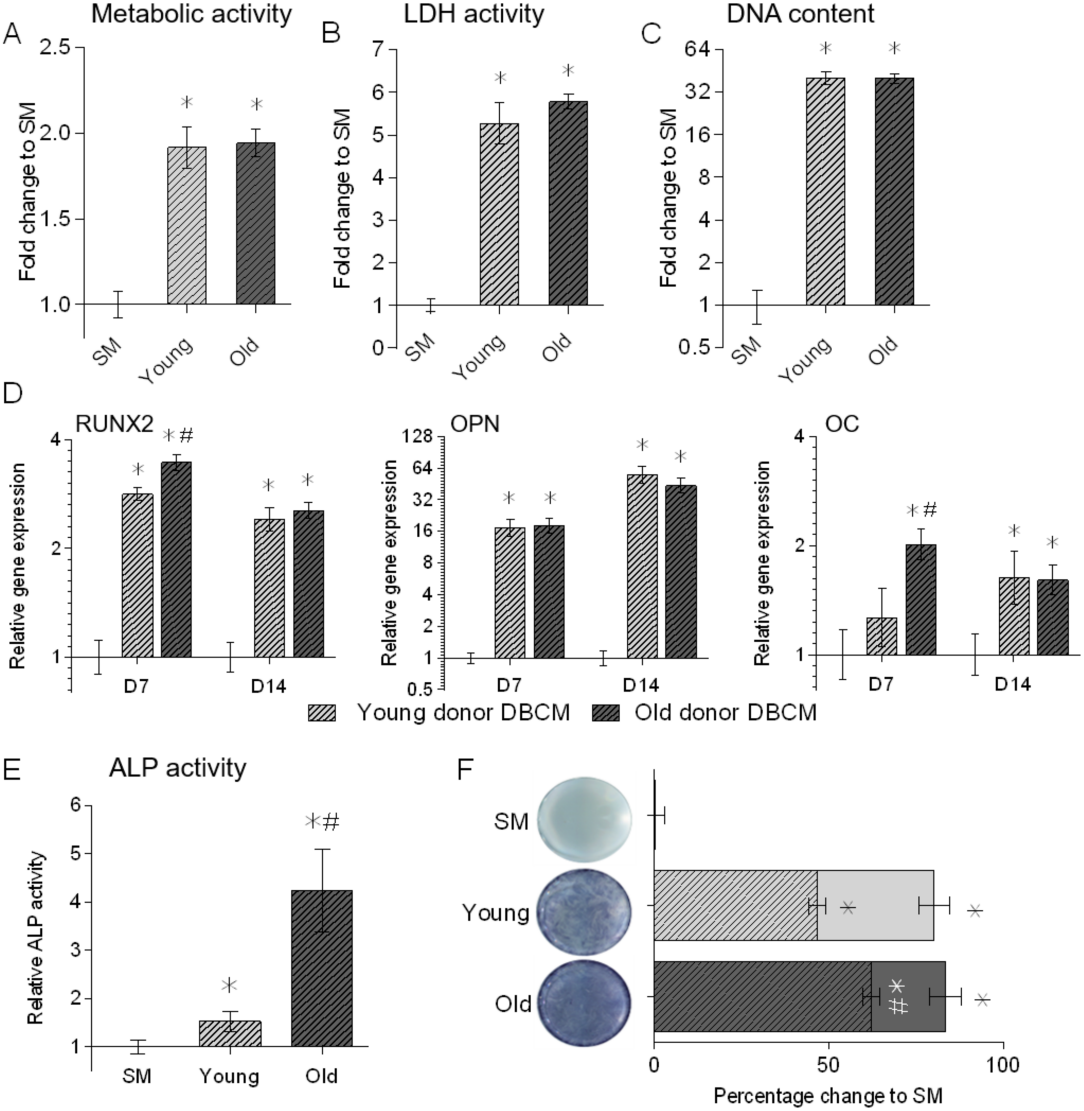
Assessment of the proliferation and osteogenic differentiation of young donor BM-MSCs (N = 3) cultured in triplicate in DBCM from young and old donors (N = 3 for each) for 14 days. Cell proliferation and viability were assessed by alamarBlue cell health indicator assessment of cell metabolic activity (a), picogreen analysis of total DNA in sample (b), and LDH assay for total live cell (c), all in comparison to SM. Relative gene expression of osteogenic markers RUNX2, OPN and OC (d) were normalised to reference gene MRPL19 and SM controls (2^-ΔΔCT^). Relative ALP enzymatic activity (e), normalised to DNA content, and Visual BCIP NBT staining of active ALP enzyme (f), displayed as quantification of area covered by stain (whole coloured bars) and staining intensity (chequered area) were both normalised to SM controls. All data is shown relative to SM control at the same time-point. Data represent mean ± SE. * indicates significant difference to day 0 (p≤0.05). # indicates significant difference between DBCM at the same time-point (p≤0.05).

Results of the conditioned media assay indicated that culture of BM-MSCs in DBCM from either young or old donors led to significant increases in cell metabolic activity (Fig 3A) and eventual total cell number (Fig 3B), with LDH assays indicating these cells were viable (Fig 3C). All BM-MSC / DBCM combinations displayed significant increases in osteogenic gene expression for RUNX2, OPN and OC (Fig 3D), as well as significantly increased ALP activity compared to standard medium controls (Fig 3E). Furthermore, cells cultured in old donor DBCM expressed significantly higher levels of RUNX2 and OC at day 7 compared to young donor DBCM culture cells, as well as significantly higher ALP activity at day 14. This difference in ALP activity was corroborated by ALP staining which demonstrated that, while there was a difference in the area stained for ALP activity, the percentage staining intensity for old donor DBCM was significantly higher than young donor DBCM (Fig 3F).

**Fig 4:**
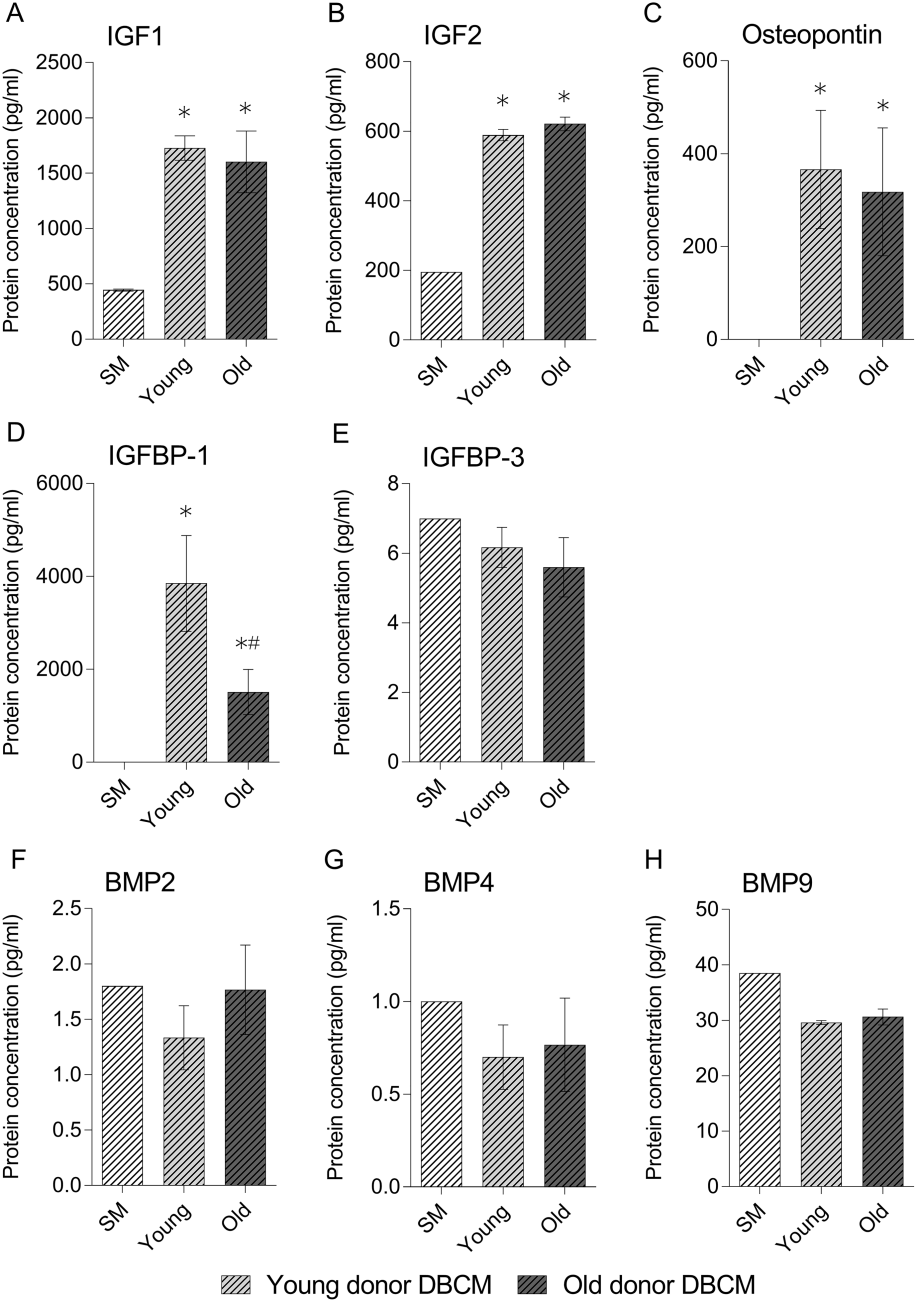
Assessment of bone growth associated growth factors contained in DBCM from young and old donors for IGF-I (a), IGF-II (b), Osteopontin (c), IGFBP-1 (d) and 3 (e) and BMP’s 2 (f), 4 (g) and 9 (h). IGF-1 and IGF-II were assessed via sandwich ELISA; all others were analysed via Luminex assay. Readings are displayed as pg/ml relative to a standard concentration curve as provided in kit. Data represent mean ± SE. * indicates significant difference to day 0 (p≤0.05). # indicates significant difference between DBCM at the same time-point (p≤0.05).

The results of the protein assays indicated there was no significant difference in the protein concentration of IGF-I, IGF-II or osteopontin (Fig 4A, 4B and 4C respectively) in DBCM produced from either young or old individuals. However, young DBCM contained a significantly higher concentration of IGFBP-1 (Fig 4D). There was no significant change in any of the BMP’s analysed in either age group DBCM compared to standard medium controls (Fig 4F, 4G and 4H respectively).

## Discussion

Biological bone grafts offer the best material for bone tissue regeneration [5]. Importantly, the number of patients requiring bone graft is increasing [1], meaning that allograft bone donated by both young, but predominantly old donors is increasingly being utilised to meet clinical demand. Decellularization techniques, which remove the cells and immunogenic material from native tissue and produce biological ECM scaffolds suitable for cell-based tissue engineering techniques [6, 7, 20], rely on the innate osteogenic qualities of the donated bone. As such, the impact of ageing on the decellularized bone scaffolds osteoinductive capacity needs to be assessed to better understand how age may affect the hosts response and its eventual clinical efficacy.

Importantly, as aging occurs, the functionality and vitality of osteoblasts also decreases [21], and changes in protein or growth factor expression incorporated in any newly synthesised bone may alter osteogenic activity of the progenitors or pre-osteogenic cells cultured upon it. Our results indicated that BM-MSCs from either cell donor age group, cultured on old donor bone, consistently expressed significantly higher levels of osteogenic genes at comparable time-points compared to those cells cultured on young donor bone. These results suggest that old donor bone was better able to support the osteogenic differentiation of BM-MSCs. In addition, although the results show that BM-MSCs from all donors underwent osteogenic differentiation, they do indicate that cells from younger donors displayed a more differentiated cell phenotype as has been previously reported [22], demonstrated by osteocalcin expression being significantly higher in young samples.

Ultimately aged bone displays changes in the microarchitecture of the ageing trabecular structure [13]. Our data clearly shows this significant change in architecture between the two age groups, most notably a decrease in connective density and increase in porosity of old donor bone compared to young, in line with the literature [23, 24]. Importantly, this difference in structure could account for the difference in osteoinductive potential, as more porous biological and synthetic materials have been demonstrated to have a greater osteoinductive capacity [20, 25]. However, although there were substantial architecture changes, there was no significant difference observed in bone mineral density, hydroxyapatite crystal structure or Ca/P ratio between the young and old bone samples. Such results indicate that there was no difference in the mineral crystallinity between the two age groups which is consistent with the literature [26], and would suggest that a change in mineral crystallinity is not responsible for the differences recorded in the osteoinductive ability between young and old donor bone.

The protein composition of bone is considered to be its most osteoinductive component, comprising of osteoconductive type I collagen and non-collagenous proteins, including encapsulated soluble bioactive factors [27, 28]. To determine if soluble factors alone could account for the osteoinductive capacity of the decellularized ECM, DBCM osteoinductive assays were undertaken. Importantly, BM-MSCs cultured in DBCM produced from either young or old bone underwent osteogenic differentiation, independent of other osteoinductive factors indicating the release of soluble osteogenic factors [29]. Additionally, BM-MSCs cultured in ODBCM displayed greater osteogenic activity as compared to YDBCM, as demonstrated by earlier expression of late stage marker OC, increased ALP activity and staining intensity. Although there have been studies comparing the relative concentrations of osteogenic growth factors and non-collagenous proteins in bone from donors of different age [30–33], they have not established whether the resulting changes affect the osteoinductive potential of the bone [34]. This study has demonstrated the effect of soluble factors from the decellularized bone in the promotion of osteogenic differentiation, additionally noting differences in the gene expression and ALP activity between the two age groups. Analysis of the protein and growth factor content of the DBCM indicated the presence of several factors including osteopontin, IGF-I and IGF-II, which have previously been identified as important for bone growth and development and which differ with age [30–34], as well as BMPs both associated (BMP 2, 9) and not associated with osteogenesis (BMP 4) [35]. Importantly, this study found no significant difference between the two age groups in the concentration of these growth factors, similar to previous studies [33]. However, interestingly there was a significant increase in IGFBP-1 content in young DBCM compared to old. IGFBP-1 is capable of binding both IGF-I and II, although it binds IGF-I with greater affinity. This binding inhibits IGF-I function by decreasing its free/bioactive levels [36]. This finding may explain the delayed osteogenic activity and lower ALP staining observed in BMMSCs cultured in young donor DBCM compared to old. IGF-I and II have been identified as an important factor is bone homeostasis, able to bind the insulin-like growth factor receptor 1 (IGFr1), enhancing BMP activation of SMAD signalling in MSCs, and thereby promoting osteogenic differentiation [37, 38]. Therefore, free IGF-I and II in the DBCM may help to promote osteogenic differentiation in BM-MSCs, although this would require further study.

To date, research into the effect of bone donor age on the materials osteoinductive potential has mainly been undertaken with demineralised bone matrix (DBM), which demonstrates different structural and biochemical characteristics to mineralised bone [39]. Though previous studies have identified no difference in the osteoinductive potential [16, 19], or clinical outcome of using differently aged donor bone [40], it is important to note that they did not include stem/progenitor cell populations, which have been proven to improve osteogenic ability and graft incorporation instead of solely relying on the healing potential of the host.

## Conclusion

Biological structures offer the best microenvironment for tissue engineering of new bone, and to the authors knowledge this is the first study undertaken to examine the effect of bone donor age utilising large, non-powdered structures of human decellularized trabecular bone, thus protecting the unique structural bone microenvironment. This study has demonstrated the ability of decellularized bone produced from young and old donors to support the osteogenic differentiation of both young and old donor cells. Significantly, the results indicate that utilising bone retrieved from aged donors is not detrimental to the differentiation of seeded cells, with seeded BM-MSCs displaying significantly higher osteogenic gene expression when cultured on old donor bone compared to those cultured on young donor bone. This has important implications for clinical use as the osteogenic capacity of a bone tissue engineering scaffold is essential in ensuring a positive host response, enabling new bone formation and osseointegration, to ensure a good clinical outcome. Indeed, bone allograft from aged donors (>70 years) is commonly used in both primary and revision arthroplasties, incorporating into the host without any deleterious effects, and displaying excellent clinical results. Importantly, our current findings provide a scientific rationale as to why old donor bone may be the most appropriate material for clinical use, and suggest that its greater osteogenic capacity may be related to a reduced IGFBP1 bioavailability in aged bone, increased porosity or alteration in scaffold architecture.

## Supporting information

S1 Table Individual data and demographies for all young and old donor μCT analysed bone samples. (XLSX)
