# Correction: Human decellularized bone scaffolds from aged donors show improved osteoinductive capacity compared to young donor bone

**DOI:** 10.1371/journal.pone.0187783

**Published:** 2017-11-02

**Authors:** Christopher A. Smith, Tim N. Board, Paul Rooney, Mark J. Eagle, Stephen M. Richardson, Judith A. Hoyland

In the Funding section, the grant numbers are missing. The grant number for the John Charnley Trust is 2435. The grant number for the MRC CIC is MC_PC_14112 v.2.
